# The Conscious Side of ‘Subliminal’ Linguistic Priming: A Systematic Review With Meta-Analysis and Reliability Analysis of Visibility Measures

**DOI:** 10.5334/joc.419

**Published:** 2025-01-07

**Authors:** David Hernández-Gutiérrez, Miguel A. Sorrel, David R. Shanks, Miguel A. Vadillo

**Affiliations:** 1 Departamento de Psicología Básica, Facultad de Psicología, Universidad Autónoma de Madrid, Spain; 2 Basque Center on Cognition, Brain and Language (BCBL), Spain; 3 Departamento de Psicología Social y Metodología, Facultad de Psicología, Universidad Autónoma de Madrid, Spain; 4 Division of Psychology and Language Sciences, University College London, UK

**Keywords:** unconscious, language, masked syntactic priming, reliability, meta-analysis

## Abstract

Research on unconscious processing has been a valuable source of evidence in psycholinguistics for shedding light on the cognitive architecture of language. The automaticity of syntactic processing, in particular, has long been debated. One strategy to establish this automaticity involves detecting significant syntactic priming effects in tasks that limit conscious awareness of the stimuli. Criteria for assessing unconscious priming include the visibility (*d*’) of masked words not differing significantly from zero and no positive correlation between visibility and priming. However, such outcomes could also arise for strictly methodological reasons, such as low statistical power in visibility tests or low reliability of dependent measures. In this study, we aimed to address these potential limitations. Through meta-analysis and Bayesian re-analysis, we find evidence of low statistical power and of participants having above-chance awareness of ‘subliminal’ words. Moreover, we conducted reliability analyses on a dataset from Berkovitch and Dehaene (2019), finding that low reliability in both syntactic priming and visibility tasks may better explain the absence of a significant correlation. Overall, these findings cast doubt on the validity of previous conclusions regarding the automaticity of syntactic processing based on masked priming effects. The results underscore the importance of revisiting the methods employed when exploring unconscious processing in future psycholinguistic research.

Language is a unique higher-order human capacity that enables conscious communication among individuals. However, certain computations sustaining linguistic processing may not require awareness of the linguistic stimulus (e.g., Axelrod et al., 2015; Berkovitch & Dehaene, 2019; Chomsky, 1980; van Gaal et al., 2014). For successful comprehension, individuals need to syntactically process the linguistic input, applying a series of rules that allow the abstract linguistic structures and dependencies between words to be understood. During typical social interactions, conversations unfold extremely rapidly (approximately 170 words per minute in English) (Tauroza & Allisond, 1990). Despite this, listeners do not perceive language comprehension as challenging, and language processing instead occurs smoothly and effortlessly, leading to the intuition that these combinatorial operations are performed automatically. Indeed, within the psycholinguistic literature, many authors have defended the automaticity of syntactic processing based not only on this intuition but also on behavioral and neuroscientific evidence (Friederici, 2011; Jiménez-Ortega et al., 2014; Pyatigorskaya, Maran, & Zaccarella, 2023; Ullman, 2001). One strategy to examine the nature of syntactic operations involves using experimental paradigms that restrict conscious processing (e.g., continuous flash suppression, e.g., Hung & Hsieh, 2015; 2021; rapid serial visual presentation, e.g., Batterink et al., 2010; masked priming, e.g., Berkovitch & Dehaene, 2019; Pyatigorskaya, Maran, & Zaccarella, 2023). If syntactic processing takes place under conditions of limited awareness in these tasks, it is taken as a demonstration of its automaticity (Maran, Friederici, & Zaccarella, 2022).

Masked/subliminal priming is one of the most popular experimental tasks (Ansorge, Kunde, & Kiefer, 2014; Kinoshita & Lupker, 2004; Kouider & Dehaene, 2007, Van den Bussche, Van den Noortgate, & Reynvoet, 2009). As observed in the following sections, a crucial step in studies using this method is to confirm that the (masked) primes were not consciously processed. Notably, experts in other cognitive domains, such as attention and learning, have recently highlighted methodological challenges in measuring consciousness, including issues related to low statistical power and the reliability of the dependent measures (Shanks, Malejka, & Vadillo, 2021; Vadillo, Konstantinidis, & Shanks, 2016). Although most of these criticisms have been directed to other experimental procedures, many of them apply to masked priming experiments just as well. Despite the widespread use of masked priming in psycholinguistics, the field has not explicitly addressed these caveats, raising concerns about the scope and limits of unconscious linguistic processing. The present study addresses this gap, focusing specifically on masked syntactic priming. To preview our results, they indicate methodological shortcomings related to low statistical power and the lack of reliability of awareness tests, undermining claims regarding subliminal processing of the primes. The findings of this study have implications for both the language and consciousness fields.

## Masked Syntactic Priming

**Figure 1:**
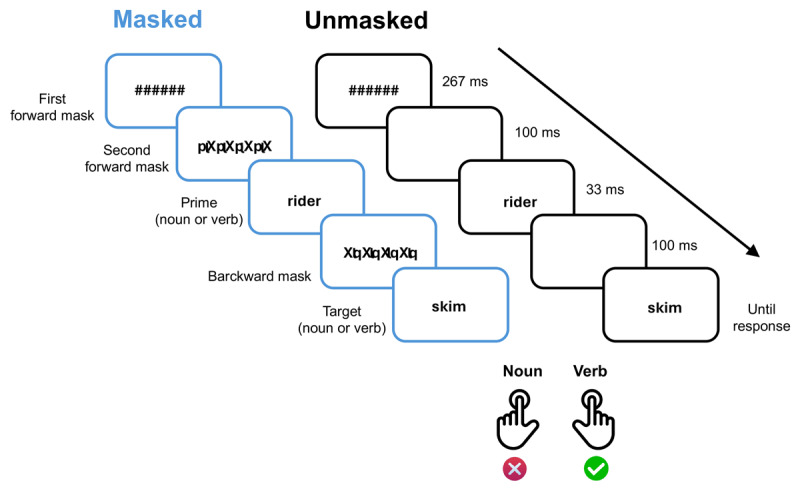
Example of stimulus presentation procedures in the syntactic priming task. *Note*. Adapted from ‘*Subliminal syntactic priming*’ by Berkovitch, L. and Dehaene, S. 2019, Cognitive Psychology, 109, p. 30. Stimulus presentation procedure for unmasked and masked trials, Experiment 1. Prime and target words were originally presented in French. In this example, the prime is a noun and the target is a verb (incongruent trial).

The priming effect can be defined as the facilitation of a response to a target stimulus (e.g., shorter reaction time in a categorization task) when presented immediately after a related prime stimulus compared to a condition where no prime is presented, or where the prime is inconsistent with the target (Kinoshita & Lupker, 2004). In the standard priming task, both the prime and the target are presented for a sufficient amount of time to be consciously processed. In contrast, masked priming tasks usually involve the brief presentation of a prime stimulus (e.g., COFFEE), followed by a target stimulus (e.g., GARDEN), to which participants are instructed to respond. In addition, a mask is usually included (e.g., ####) either before (forward masking) and/or after the prime (backward masking) with the aim of preventing conscious awareness of the prime (Figure 1).

Various linguistic properties can be primed, with semantic priming being the most commonly used, where both the prime and the target words are semantically related (e.g., DOCTOR-NURSE; for a review, see Van den Bussche, Van den Noortgate, & Reynvoet, 2009). Other studies have focused on phonology (e.g., Rastle & Brysbaert, 2006) or syntax (e.g., Berkovitch & Dehaene, 2019). In syntactic priming, a word primes a target word that shares a similar syntactic property, such as belonging to the same syntactic category (e.g., verb-verb, such as RUN-CARRY) or the same morphosyntactic gender or number (e.g., plural-plural, such as HATS-APPLES). If the participant’s task is to judge whether the target word is a noun or verb, faster RTs (i.e., priming) might be observed when the prime (a different word) and target are from the same category than from different categories. Such a priming effect would imply that the prime triggered some aspect of syntactic processing which facilitated processing of the target.

To ensure that the linguistic priming effect observed in these tasks is unconscious, many experiments include a visibility test to evaluate participants’ awareness of the primes. However, there has been inconsistency in the inclusion of visibility tests, and discrepancies exist in the methods used to assess prime visibility (Merikle & Reingold, 1992). On the one hand, some experiments collect subjective measures, asking participants whether they saw the primes consciously (e.g., Jiménez-Ortega et al., 2014; 2021). Unfortunately, subjective reports have faced strong criticism for potential bias (Dienes, 2004; Eriksen, 1960). Participants might simply be reluctant to report that they perceived something consciously unless they are very sure of the presence and nature of the prime. On the other hand, an alternative approach employs objective measures, based on participants’ performance. In this case participants are simply asked to categorize the masked primes (e.g., noun or verb) in a forced-choice test. Experimenters can then use the Signal Detection Theory (SDT) framework (Green & Swets, 1966; Hautus, MacMillan, & Creelman, 2022) to calculate participants’ sensitivity or *d*’, considering both hits and false alarms – a method we will focus on in the present paper. Alternative objective procedures also include fitting psychophysical functions and analyzing their thresholds and slopes to assess conscious processing (Kiefer & Kammer, 2017; 2024).

Once the visibility measures have been collected, researchers employ various analytic procedures to demonstrate that a syntactic priming effect has occurred unconsciously. In the case of subjective reports, participants should indicate that they have not seen or recognized the primes (e.g., Jiménez-Ortega et al., 2014; 2021). For objective procedures, researchers can rely on different strategies to reach this conclusion (Shanks, Malejka, & Vadillo, 2021). First, participants’ accuracy in the visibility task should not be above chance. In this regard, researchers usually make use of a frequentist approach and standard null hypothesis significance testing (NHST) to test the null hypothesis that the masked primes were not visible. Second, the effect size of priming performance should not correlate with visibility scores. Hence, if syntactic priming depends on awareness of the syntactic properties present in the masked prime words, the higher the visibility, the larger the priming effects. Furthermore, in instances where these analyses fail to produce the anticipated null outcomes, researchers often resort to post-hoc elimination of participants or trials in which conscious awareness of the primes is demonstrated—a procedure that may be problematic due to regression to the mean (Dienes, 2024; Shanks, 2017).

A recent study that follows this rationale in the context of masked syntactic priming was conducted by Berkovitch and Dehaene (2019). Part of our work in the present article involves a more in-depth analysis of their dataset. Therefore, a detailed examination of their study is provided to illustrate these strategies and set the scene for our follow-up analyses. However, it is important to note that this reasoning has been extensively pursued across studies in other linguistic and cognitive domains.

## Berkovitch and Dehaene (2019) as a Case Study

Berkovitch and Dehaene (2019) reported five experiments to investigate syntactic priming. In each experiment, a syntactic characteristic shared between a prime and a target word was intended to induce conscious or unconscious priming, depending on the masked or unmasked presentation of the primes (see Figure 1). They explored different types of syntactic priming in each experiment, with a similar procedure across all five. Taking Experiment 1 as an example, where grammatical category was primed, Berkovitch and Dehaene used nouns and verbs (in French) as primes and targets. Each trial involved presenting the prime word followed by the target word, and participants had to perform a grammatical categorization task, making a noun/ verb judgment on each trial. Participants were instructed to attend only to the target word and to ignore other stimuli. If the grammatical category was primed (e.g., RIDER – CASE), the presentation of a target word of the same category as the prime (congruent trial: noun-noun, verb-verb) would result in shorter reaction times compared to pairs of words from different categories (incongruent trials: noun-verb, verb-noun). There was a total of 240 masked trials and 240 unmasked trials, all randomly presented in 8 blocks of 60 trials.

To analyze the syntactic priming effect, Berkovitch and Dehaene performed a repeated-measures ANOVA on each experiment, incorporating the factors of Visibility, Category of the Prime (or Prime Number in Exp. 5), and Category of the Target (or Target Number in Exp. 5). Our focus is on the priming effect in the masked condition, summarized in Table 1. Each effect is statistically significant, with faster responses in the congruent than incongruent condition.

**Table 1:** Experimental and statistical information of the study performed by Berkovitch and Dehaene (2019).


		VISIBILITY TASK		MASKED PRIMING TASK		
			
EXPERIMENT	*n*	# TRIALS	VISIBILITY (*d*’)	# TRIALS	PRIMING (ms)	PEARSON *r*

1	16	60	0.03	240	6*	–0.5*

2	19	40	–0.24*	240	7*	N.R.

3	16	60	0.12	240	7*	N.R.

4	24	64	0.21*	480	5*	N.R.

5	24	64	0.07	480	17***	N.R.


*Note*. The values included in this table correspond to those reported by Berkovitch and Dehaene (2019), *p < 0.05, ***p < 0.001, N.R. = Not Reported.

At the conclusion of each experiment, participants undertook a visibility task, consisting of an objective forced-choice test with variable numbers of masked trials, as reported in Table 1. The goal was to ascertain whether the syntactic characteristic tested (e.g., grammatical category in Experiment 1) had been unconsciously processed in the masked condition. In this task, participants were informed in advance that a prime word preceded every target word. Similar to the experimental task, they were asked to make a categorization decision, but in this case, the judgment pertained to the prime word rather than the target. Participants were explicitly told that only accuracy, not response speed, was relevant, and they were required to respond even if they lacked confidence in the correct choice. The discrimination index *d*’ was employed to assess visibility for each participant.

Once the syntactic priming and visibility scores were computed for each participant, the authors followed the strategies previously outlined to demonstrate the unconscious nature of the effects in the masked condition. Therefore, a *t*-test was first conducted to assess the hypothesis that visibility *d*’ was not significantly different from zero. As detailed in Table 1, results for the visibility task in the masked condition across the five experiments revealed that only one (Exp. 4) out of five effects was both significant and in the right direction.

After obtaining the performance measures of subliminal syntactic priming and visibility in the masked condition, the authors correlated these measures. They only found a significant (but negative) correlation in Experiment 1 (see Table 1). For the other four non-significant effects the authors reported the statistical comparison but not the correlation value (all *p*s between 0.23 and 0.74).

This combination of results (i.e., chance-level *d*’ and no significant correlation between *d*’ and priming in most experiments) led the authors to assert that syntactic properties such as number and grammatical category can be processed unconsciously. However, this conclusion hinges on the assumption that the measures obtained across the five experiments are meaningful and that statistical power was sufficiently high. Hence, did Berkovitch and Dehaene (2019) indeed identify subliminal effects?

## Potential Methodological Limitations of Research on Unconscious Linguistic Priming

To this point we have presented some of the strategies that are repeatedly followed in subliminal linguistic priming studies to demonstrate unconscious priming, but to what extent can we trust the logic of these analyses? In a recent publication, Shanks, Malejka, and Vadillo (2021) conducted a thorough examination of the various arguments that have been used to infer that different cognitive processes (not specifically linguistic) are unconscious. They pointed out several methodological problems that complicate the assessment of unconscious mental processes and proposed potential solutions. Here, we will focus particularly on two of them that directly relate to the previously given arguments.

The first issue regards the pattern of null results in the visibility task combined with significant masked priming in the experimental task. The concern with this pattern is that, within frequentist statistical testing, a nonsignificant result does not necessarily mean that the null hypothesis is true. This outcome could be attributed to factors such as low statistical power (given that sample sizes are usually quite small and, furthermore, the visibility task usually includes fewer trials than the priming task) or noisy measures. For instance, in the domain of unconscious contextual cueing, Vadillo, Konstantinidis, and Shanks (2016) highlighted that most studies had a median of only 16 subjects, resulting in very wide confidence intervals around the estimated level of awareness. A possible solution to this pitfall is to conduct a meta-analysis to achieve higher power in the visibility task. Another potential remedy involves using Bayes factors (BF) to assess support for the null hypothesis. This problem can also be ameliorated by increasing the number of trials to boost statistical power (Dienes, 2014).

The second issue regards not finding a significant correlation between the effect sizes of the experimental task (e.g., masked syntactic priming) and the visibility task. As well as again reflecting a null result, the problem with this pattern lies in the fact that it assumes that both variables are measured without measurement error, but this is an unlikely assumption, leading to correlation attenuation. In fact, many experimental tasks that intend to measure unconscious processes have disappointingly low reliabilities (e.g. Vadillo et al. 2022, 2024; Yaron et al. 2024). Under this scenario, it is not surprising that two dependent measures fail to correlate significantly, even if at the latent level they are related. While the scientific community has increasingly recognized the risks of using unreliable measures, and researchers are more frequently addressing this issue, the use of the null correlation strategy remains common, with important consequences. Therefore, it is advisable to always report reliability estimates to ensure the correct interpretation of the scores, and the correlations derived from them.

## The Present Study

Given the major implications of these potential methodological limitations, this study explores the pitfalls mentioned above in awareness measures within studies investigating unconscious syntactic processing through masked priming. Specifically, our focus centers on the zero visibility and null correlation arguments typically employed to support the existence of unconscious processing (Shanks, Malejka, & Vadillo, 2021).

Firstly, to investigate the claim of non-significant visibility at the group level, we conduct a systematic review and meta-analysis of experiments that have explored syntactic processing using the masked priming task. The review follows the PRISMA 2020 guidelines (Page et al., 2021). It is important to note that in the meta-analysis, we analyze the visibility test rather than the experimental priming task. Our interest lies not directly in the priming effect (which is usually robust and statistically significant in these studies), but in the degree of awareness of the masked words (for meta-analysis on linguistic priming effects, see Van den Bussche, Van den Noortgate, & Reynvoet, 2009). We select studies that employ objective measures of visibility and report a *d*’, which serves as our parameter of interest. This meta-analysis is essential to assess the overall visibility of masked words with high precision. By aggregating across studies, we aim to achieve high statistical power, addressing statistical issues associated with small samples of participants and numbers of trials. Considering the highlighted limitations, we anticipate the meta-analysis will reveal a significant average visibility effect. Furthermore, power analyses are performed on each study, and we compute the average sample size necessary to achieve 80% power.

Secondly, we conduct a Bayesian analysis on each of the experiments previously included in the frequentist meta-analysis to weigh the strength of the evidence for participants’ awareness or the absence thereof. Subsequently, we combine all the studies in a single Bayesian meta-analysis to overcome potential disparities in results found between them. In line with our previous predictions, we expect to find evidence of conscious processing of masked stimuli in most studies, particularly when results are combined.

To address the possible attenuation in the non-significant correlation between priming and awareness, we calculate the split-half reliability of both the priming and visibility tests in the five experiments conducted by Berkovitch and Dehaene (2019) as an illustrative dataset, generously provided by them. Our primary aim with this procedure is to determine whether the null correlations reported in each of the experiments could be better explained by the low reliability of the masked priming and awareness measures. Considering results from other studies that employ objective measures (e.g., Vadillo et al., 2022, Yaron et al., 2024), we anticipate that the reliability of these measures will be too low to allow for the kind of correlational analyses typically conducted with them. As previously explained, correlation coefficients between two different measures cannot be expected to be high if the measures are noisy.

Next, we conduct a psychometric meta-analysis to assess, in a single analysis, the correlation between priming and visibility after taking measurement and sampling error into account. This method, already employed in other studies investigating the limitations of unconscious processing (e.g., Vadillo et al., 2022; 2024), offers a comprehensive approach to evaluate correlations between noisy measures (Wiernik & Dahlke, 2020).

Finally, we perform a sensitivity comparison in Berkovitch and Dehaene’s (2019) experiments to directly contrast masked priming and visibility effects (Meyen et al., 2022). This method avoids common fallacies present in *standard reasoning* for inferring unconscious processing. For instance, it addresses the misconception that sensitivity to masked words fully accounts for the masked priming effect or the assumption of unconscious processing when *d*’ in the visibility test is at chance but there is a significant masked priming effect. Typically, masked priming and visibility measures cannot be directly compared, as the former is a continuous metric (reaction time) and the latter a binary metric (correct, incorrect). This often leads to performing separate *t*-tests on each task. Moreover, certain properties of the priming measure can facilitate a significant statistical effect compared to the visibility measure. To infer unconscious processing, we transform priming reaction times into *d*’_priming_, allowing for a comparison of sensitivity to the masked words in both tasks (Meyen et al., 2022, 2024).

To the best of our knowledge, this study marks the first in-depth examination of visibility data within the masked syntactic priming literature and, more broadly, in linguistic masked priming studies.

## Method

### Literature Search

In January 2024 we conducted a search in PubMed using the search string ‘(syntax or syntactic or morphosyntactic) and (masked or subliminal or unconscious or automatic)’.^1^ Van den Bussche, Van den Noortgate, and Reynvoet’s (2009) meta-analysis on subliminal semantic priming guided our criteria for selecting studies, which we adapted to syntactic priming and aligned with the goals of our research. Accordingly, the following criteria were employed to determine the eligibility of studies for inclusion in the meta-analysis. Firstly, only studies focusing on masked syntactic priming were considered. Other methods for investigating unconscious or automatic syntactic processing, such as speech masking, competing speech perception, continuous flash suppression, or binocular rivalry, were excluded. This decision is motivated by the popularity of the masked priming method in psycholinguistics and ensures the inclusion of a set of studies that are highly comparable. Secondly, only studies where the target and prime were syntactically related were selected. Thirdly, studies were included if primes were intentionally presented unconsciously. Specifically, visual presentation of the primes lasted for 100 ms or less and was accompanied by a mask, following the typical procedure of masked priming. Consequently, participants were not instructed to perform any task involving the primes during the priming task.^2^ Fourthly, studies in which the visibility of the primes was assessed using the discrimination index *d*’ were selected. Studies were excluded if they aimed to study subliminal language but failed to assess the visibility of the primes or used only subjective measures (for which *d*’ cannot be calculated). Fifthly, we only considered studies reporting sufficient statistical information for the necessary analyses.

**Figure 2:**
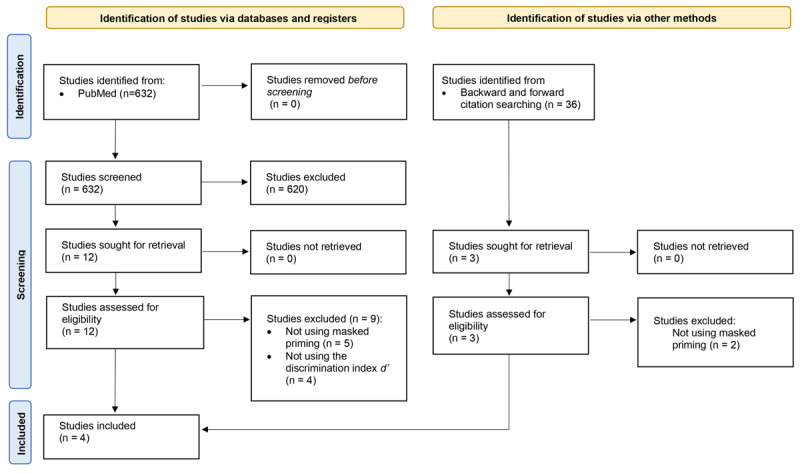
PRISMA 2020 flow diagram of the literature search strategy (Page et al., 2021).

We initially identified 632 studies from the PubMed search (see Figure 2). After an initial screening based on their titles and abstracts, 620 studies were excluded either due to the research topic or for not meeting our selection criteria. The large number of exclusions was due to the broad range of keywords used, as the masked priming paradigm has not always been labeled consistently. While this resulted in a highly unspecific search, it ensured that relevant studies were included. Most of these exclusions were due to studies focusing on topics unrelated to linguistic processing, while fewer were excluded for not meeting our selection criteria, particularly for not focusing on syntactic priming. The remaining 12 studies were eligible for full-text review. Once completed, 5 studies were excluded for not utilizing masked priming (Batterink & Neville, 2013; Hasting & Kotz, 2008; Lau, Liang, & Leung, 2017; Mirault & Grainger, 2020; Pulvermüller & Shtyrov, 2006) and 4 were excluded for not using Signal Detection Theory to assess prime visibility (Afonso et al., 2014; Jiménez-Ortega et al., 2014; 2017; 2021). This left us with 3 studies that met all our criteria. Subsequently, we conducted an updated search, including forward and backward citation searching on these 3 studies. After reviewing 3 potential candidates, 2 were excluded for not assessing syntactic priming (Namdari et al., 2021, Peel, Royals, & Chouinard, 2022), and 1 was selected. This process resulted in the inclusion of 4 studies in the meta-analysis, encompassing a total of 16 individual experiments (n = 328): Ansorge et al. (2013), Iijima and Sakai (2014), Berkovitch and Dehaene (2019), and Pyatigorskaya, Maran, and Zaccarella (2023).

### Frequentist Meta-Analysis of Prime Visibility

The meta-analysis was performed to test whether participants in masked syntactic priming studies saw the ‘subliminal’ stimuli. Therefore, we were not interested in the experimental task but in the accompanying visibility tests. Those studies that met the previous criteria were included in the meta-analysis. The meta-analysis was performed using the ‘*metafor*’ package for R (Viechtbauer, 2010).

We conducted two meta-analyses of effect estimates, one based on *d*’ and the other on Cohen’s *d*_z_. This second measure can be obtained when the *t*-values of the comparison between supraliminal and subliminal stimuli are available, as is the case here. To obtain this value, it must be divided by the square root of the sample size. Additionally, the *F*-value of a within-subjects comparison can be converted to a *t*-value and then used to calculate *d*_z_ (Rosenthal, 1991). The variance of *d*_z_ was computed for each study according to the following formula:

\[{V_i}\; = \;\frac{1}{{{N_i}}}\; + \;\frac{{{d_i}}}{{2{N_i}}}\]

where *i* represents each experiment, *N* the sample size, and *d* the effect size (Cumming, 2013).

### Bayesian Reanalysis of Individual Studies and Meta-Analysis

The primary advantage of Bayesian analyses in this context is their ability to assess the degree to which the evidence supports the null hypothesis (Dienes, 2014). This is crucial in the realm of unconscious processing because one common argument used to assert that priming is unconscious is that, since *d*’ typically does not significantly differ from zero, it is presumed that the null hypothesis holds true. However, within the frequentist framework, this interpretation is unjustified.

We utilized the R library ‘*BayesFactor*’ to compute Bayes Factors (BFs) using the values of the *t* statistic from each experiment (Morey & Rouder, 2011). BFs were calculated to assess the evidence for H_1_ over H_0_ (BF_10_). Subsequently, we conducted a Bayesian meta-analysis, following Morey and Rouder (2011), also using the R library ‘*BayesFactor*’. In this case, the *t* statistics of each experiment, along with their sample sizes, were combined in a single meta-analysis. This procedure provides a Bayes factor against H_0_ (*d*_z_ = 0).

### Split-Half Reliability of Subliminal Syntactic Priming Task and Visibility Measures

These analyses were conducted using only the dataset from Berkovitch and Dehaene (2019) to test whether the null correlation between priming and awareness was potentially a result of correlation attenuation due to the use of unreliable measures. The five experiments included in their study were reanalyzed to calculate the split-half reliability of both the masked priming and visibility measures.

Before conducting the reliability analysis, we removed outliers using the approach described by Berkovitch and Dehaene (2019). Within both the priming task and the visibility task, we performed a split-half correlation to estimate the reliability of the measures, as recommended for behavioral experiments (Parsons, Kruijt, & Fox, 2019). This involved splitting the total set of trials into two random halves, computing the dependent measures separately on each half, and then correlating the values in the two halves across participants. To prevent an imbalance in the split halves the data were stratified. Stratifying into blocks ensured that each half contained a similar number of elements from each block, thereby controlling for potential effects of fatigue or learning (Pronk et al., 2021). Additionally, we stratified by category of the prime (e.g., verb, noun), category of the target (e.g., verb, noun), and congruency of the trial (congruent, incongruent). To mitigate the effects of sampling error in this procedure, we repeated the analyses 10,000 times and calculated the mean split-half correlation. A drawback of this permutation procedure is that the resulting correlation is attenuated due to the reduced number of trials in each group compared to the total number of trials, impacting the reliability coefficient (Nunnally, 1970). To correct for this, we employed the Spearman-Brown prediction formula: r*_xx_ = 2r_xx_ / (1 + *r_x_*_x_) (Brown, 1910; Spearman, 1910). r*_xx_ indicates the variance of the observed scores explained by the true scores, that is, an estimate of the part not due to measurement error.

An assumption of this method is that the split-halves are parallel forms, measuring the same construct with the same reliability and having equal means and variances. Therefore, negative values are not interpretable for *r_xx_* (and consequently, neither for r*_xx_). However, as shown in the *Results* section, the computation of *r*_xx_ for both tasks resulted in negative values in several iterations. Following the suggestion by Parsons, Kruijt, and Fox (2019) and Pronk et al. (2021), we treated them as zero. Therefore, the mean r*_xx_ values, based on 10,000 permutations, were computed applying the Spearman-Brown correction and considering negative values as zero. Note that, if anything, this procedure can result in a positive bias in the estimation of reliabilities.

### Psychometric Meta-Analysis

A psychometric meta-analysis attempts to estimate the effect sizes of different studies removing any bias caused by low reliability of measures or small participant samples (Schmidt, 2010; Schmidt & Hunter, 2014; Vadillo et al., 2022; Wiernik & Dahlke, 2020). In this study, we conducted a psychometric meta-analysis on the correlations between masked priming and visibility in the five experiments included in Berkovitch and Dehaene (2019). Every statistic was recalculated from the raw dataset. The psychometric meta-analysis utilized the ‘*psychmeta*’ library for R (Dahlke & Wiernik, 2019).

### Sensitivity Comparison Between Masked Syntactic Priming and Visibility

Comparing the magnitude of the priming effect and the visibility measure is not directly possible because they are based on different measurement scales. Therefore, to compare their sensitivity, reaction times of the priming effect were recalculated as *d*’_priming_ using the method described by Meyen et al (2022). This allows considering reactions times as a binary response by splitting them at the median. The procedure was performed individually on each of the five experiments as well as overall. If there is unconscious syntactic processing, sensitivity to masked primes in the masked priming task should be significantly higher than sensitivity in the visibility task (*d*’_priming_ > *d*’).

## Results

### Frequentist Meta-Analysis

**Figure 3:**
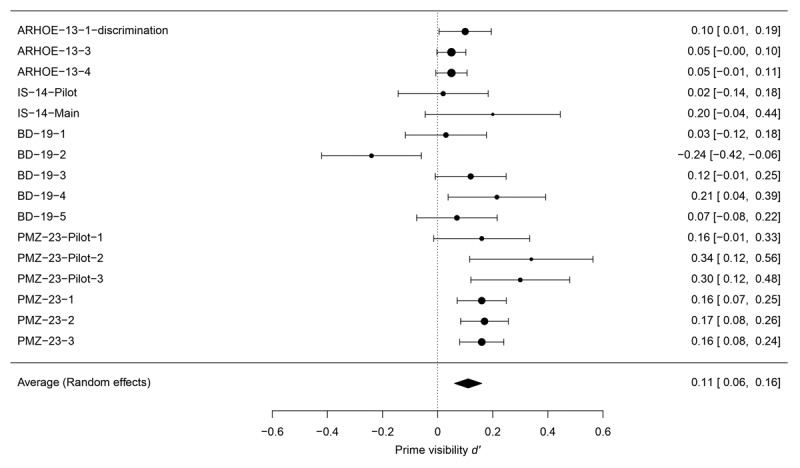
Forest-plot of the *d*’ meta-analysis. Experiments included are coded in the left column according to the initial letters of the names of the authors, followed by the last two digits of the year of publication, and the number of the experiment within the study, or its category (Pilot, Main).

Our analysis of *d*’ (see Figure 3) across the 16 experiments revealed that visibility consistently (with only one exception) exceeded zero, with a calculated mean *d*’ of 0.11 (95% CI [0.06, 0.16], *z* = 4.38, *p* < .001). Notably, the analysis unveiled a significant degree of heterogeneity among these studies (*Q* = 40, *p* < 0.001). Additionally, results showed a true variability (*I*^2^) of 66.59%, indicating inconsistency among the studies included in the meta-analysis. Despite the fact that the null effect was not rejected in 9/16 individual experiments (as can be seen from the CIs in the rightmost column of Figure 3), it is robustly rejected when the data are aggregated.

We must acknowledge the potential limitation that the 16 experiments included in the meta-analysis originate from only four studies, which may introduce bias. However, we suspect that if such a bias were present, it would likely have favored results supporting unconscious syntactic processing, contrary to the findings of our analysis.

**Figure 4:**
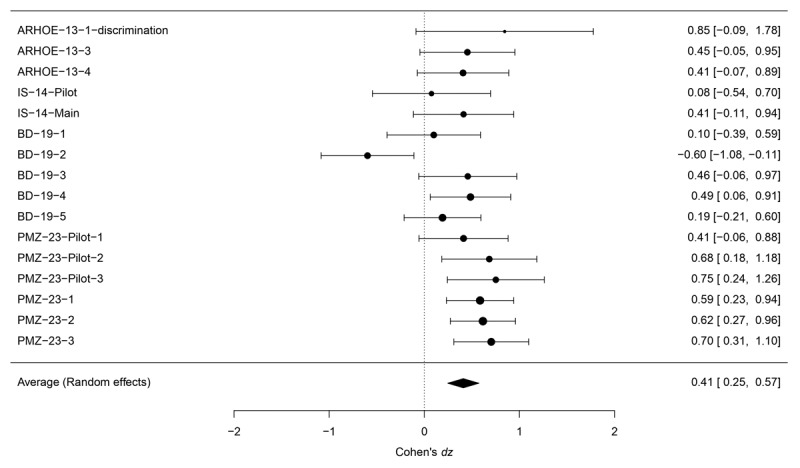
Forest plot representing the results of the Cohen’s *d_z_* meta-analysis. Experiments included are coded in the left column according to the initial letters of the names of the authors, followed by the last two digits of the year of publication, and the number of the experiment within the study, or its category (Pilot, Main).

A similar meta-analysis on Cohen’s *d_z_* (see Figure 4) yielded an average effect size of *d*_z_ = 0.41, 95% CI [0.25, 0.57], *z* = 4.92, *p* < 0.001. This analysis also revealed significant heterogeneity across the effect sizes (*Q* = 28.462, *p* = 0.019) and showed that the percentage of true variability is *I*^2^ = 47.89%.

Additionally, we calculated the power of the experiments to detect a Cohen’s *d*_z_ similar to those found in the above meta-analyses. This computation was performed using the ‘*pwr*’ R package, following the approach outlined by Cohen (1988). We also estimated the average sample size necessary to achieve a power of 80%, considering the mean effect size found in the Cohen’s *d_z_* meta-analysis. Results indicate that statistical power varies from a minimum of 0.13 to a maximum of 0.70 among the sixteen experiments (see Table 2 for a summary of all the parameters). Considering the average effect size of the visibility test *d_z_* = 0.41, the estimated sample size necessary to obtain 80% power is 49 participants, exceeding the number included in every individual experiment.

**Table 2:** Values for the statistical parameters of each study included in the meta-analysis.


STUDY	EXPERIMENT	*n*	*t*	*d*’	STANDARD ERROR	COHEN’S *d_z_*	POWER	BF_10_

ARHOE-13	ARHOE-13-1	6	2.07	0.100	0.048	0.845	0.131	1.335

	ARHOE-13-3	17	1.87	0.050	0.027	0.453	0.356	1.028

	ARHOE-13-4	18	1.73	0.050	0.029	0.408	0.376	0.841

IS-14	IS-14-Pilot	10	0.24	0.020	0.083	0.076	0.214	0.316

	IS-14-Main	15	1.60	0.200	0.125	0.413	0.316	0.745

BD-19	BD-19-1	16	0.40	0.030	0.075	0.100	0.337	0.274

	BD-19-2	19	– 2.60	– 0.240	0.092	– 0.596	0.395	3.190

	BD-19-3	16	1.83	0.120	0.065	0.457	0.337	0.985

	BD-19-4	24	2.38	0.215	0.090	0.486	0.487	2.197

	BD-19-5	24	0.94	0.070	0.074	0.192	0.487	0.319

PMZ-23	PMZ-23-Pilot-1	19	1.80	0.160	0.089	0.413	0.395	0.912

	PMZ-23-Pilot-2	19	2.98	0.340	0.114	0.684	0.395	6.248

	PMZ-23-Pilot-3	19	3.28	0.300	0.091	0.752	0.395	10.868

	PMZ-23-1	36	3.52	0.160	0.045	0.587	0.668	26.416

	PMZ-23-2	39	3.85	0.170	0.044	0.616	0.705	64.115

	PMZ-23-3	31	3.92	0.160	0.041	0.704	0.600	63.534


*Note*. Experiments included are coded in the second left column according to the initial letters of the names of the authors (same as the Studies, in the leftmost column), followed by the last two digits of the year of publication, and the number of the experiment within the study, or its category (Pilot, Main). Pyatigorskaya, Maran, and Zaccarella (2023) reported *t*-values against 0.12 in their three main experiments. Therefore, we calculated the *t*-values against zero by dividing the *d*’ by the standard error.

### Bayesian Reanalysis of Individual Studies and Meta-Analysis

BFs resulting from the Bayesian analysis can be found in Table 2. These BFs show the evidence for the alternative hypothesis (H_1_) that visibility is not 0 against the null hypothesis (H_0_) that visibility of the masked primes is 0. Our analysis reveals a diverse landscape of evidence regarding the competing hypotheses, although the strength of the evidence is not uniformly distributed between them. Among the examined studies, nine demonstrate evidence in favor of H_1_, with varying degrees of strength. Specifically, three studies provide anecdotal evidence (BF_10_ ranging from 1.028 to 2.197), suggesting a preference for visibility over subliminal processing without overwhelming support. Additionally, two studies offer moderate evidence of visibility of masked words (BF_10_ = 3.190 and BF_10_ = 6.248), and four studies provide strong evidence (BF_10_ ranging from 10.868 to 63.534). Conversely, seven studies present evidence favoring H_0_, with differing strengths. Four of them suggest anecdotal preference for subliminal over conscious processing (BF_10_ ranging from 0.745 to 0.985). Furthermore, three studies offer moderate evidence against the visibility of the masked words (BF_10_ ranging from 0.274 to 0.319), supporting unconscious processing.

The results from the Bayesian meta-analysis, which aggregated all the data in a single analysis, are particularly useful in this case considering the variety of individual results. This resulted in a BF_meta_ of 1.67 × 10^10^, which indicates extreme evidence for conscious processing of masked words over subliminal processing.

### Reliability of Subliminal Syntactic Priming and Visibility Measures

**Figure 5:**
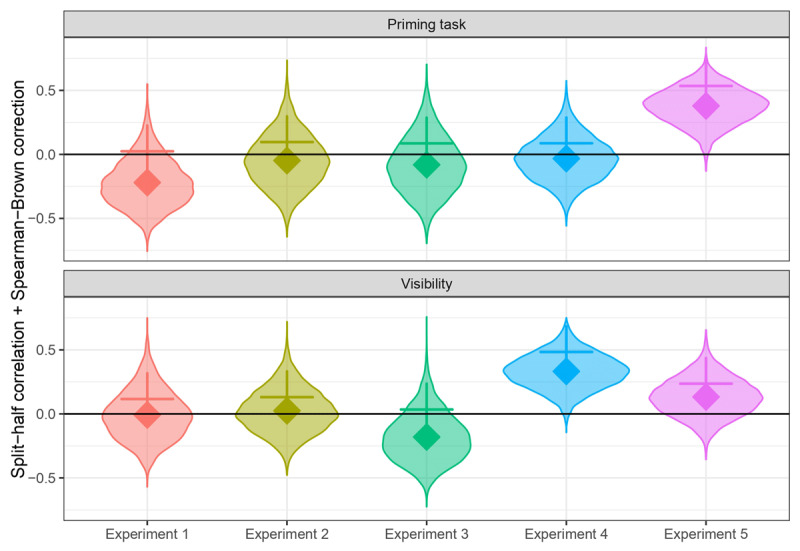
Violin plots representing the distribution of permuted reliability scores computed as split-half correlations (*r_xx_*) for priming and visibility tasks across the five experiments conducted by Berkovitch and Dehaene (2019). Diamonds indicate the mean *r_xx_* for each experiment. Crosses represent the corresponding mean Spearman-Brown correction (r*_xx_) with negative values treated as zero.

The *r_xx_* estimates were calculated for both the priming and visibility tasks across each of the five experiments conducted by Berkovitch and Dehaene (2019). Results are plotted in Figure 5. The notably low and negative split-half reliability values observed prompt reflection on the noisy measures employed. To correct the split-half coefficients, we applied the Spearman-Brown correction for attenuation after setting the negative scores to zero to avoid exacerbating the negativity (Pronk et al., 2021). Then we computed the mean r*_xx_ considering both the zero and the positive values. Remarkably, the highest reliability for priming after this adjustment is only 0.53 and the highest for visibility is only 0.49. All other reliabilities are lower than these values. These findings raise concerns regarding the consistency and stability of the priming and visibility scores.

### Psychometric Meta-Analysis

**Figure 6:**
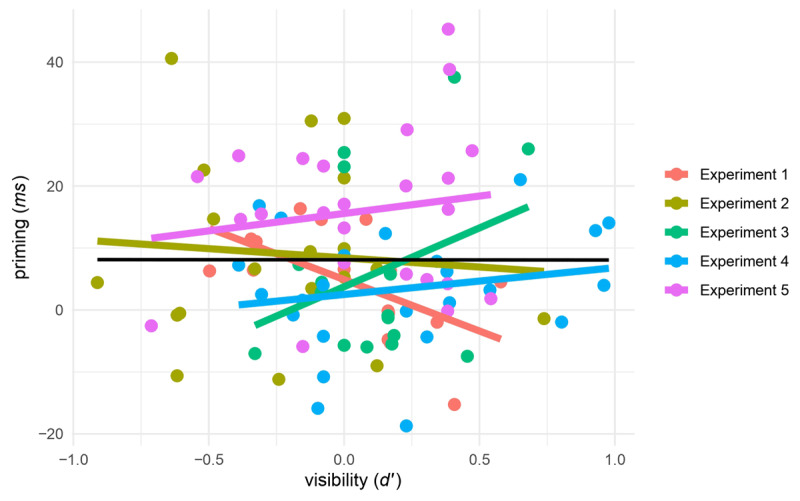
Representation of the correlation between the performance in the masked priming task and the visibility task across the five experiments. Each dot represents an individual participant, with different colors referring to each of the five experiments. The colored lines represent the trend of the correlation for each experimental sample. The black line depicts the trend for the five experiments combined.

Results of the psychometric meta-analysis yielded a mean correlation of 0.388 and a confidence interval spanning the whole range of valid values for a correlation coefficient [–1, 1]. Since the findings did not reach significance, we cannot dismiss the possibility that the correlation between priming and visibility may be as small as zero. However, considering the confidence interval, we also cannot rule out the hypothesis that the correlation could be as high as 1. Clearly, even with a total sample size of 99 participants, Berkovitch and Dehaene’s experiments are ultimately nondiagnostic about the magnitude of the priming-awareness correlation. Figure 6 represents this correlation for each experiment.

### Sensitivity Difference Between Priming and Visibility

The priming effects in Berkovitch and Dehaene’s experiments were on the order of a few milliseconds (Exp. 1: 4.39 ms; Exp. 2: 9.09 ms; Exp. 3: 5.91 ms; Exp. 4: 3.40 ms; Exp. 5: 15.9 ms) while the visibility effects went up to 0.22 *d*’ units.^3^ These are of course incommensurable measures that cannot be directly compared, hence we cannot in any sense ask whether the magnitudes of the priming effects are larger (or smaller) than the magnitude of the visibility measures. Meyen et al. (2022) introduced a useful method by which implicit (priming) and explicit (visibility) measures can be directly compared. This requires dichotomizing the priming measure to yield counts of ‘hits’ and ‘false alarms’ that can then be entered into the standard formula for calculating *d*’. Full details are provided by Meyen et al., but in essence a median split is applied such that a response to a congruent word that is faster than the median is classified as a hit while a response to an incongruent word that is faster than the median is classified as a false alarm.

**Figure 7:**
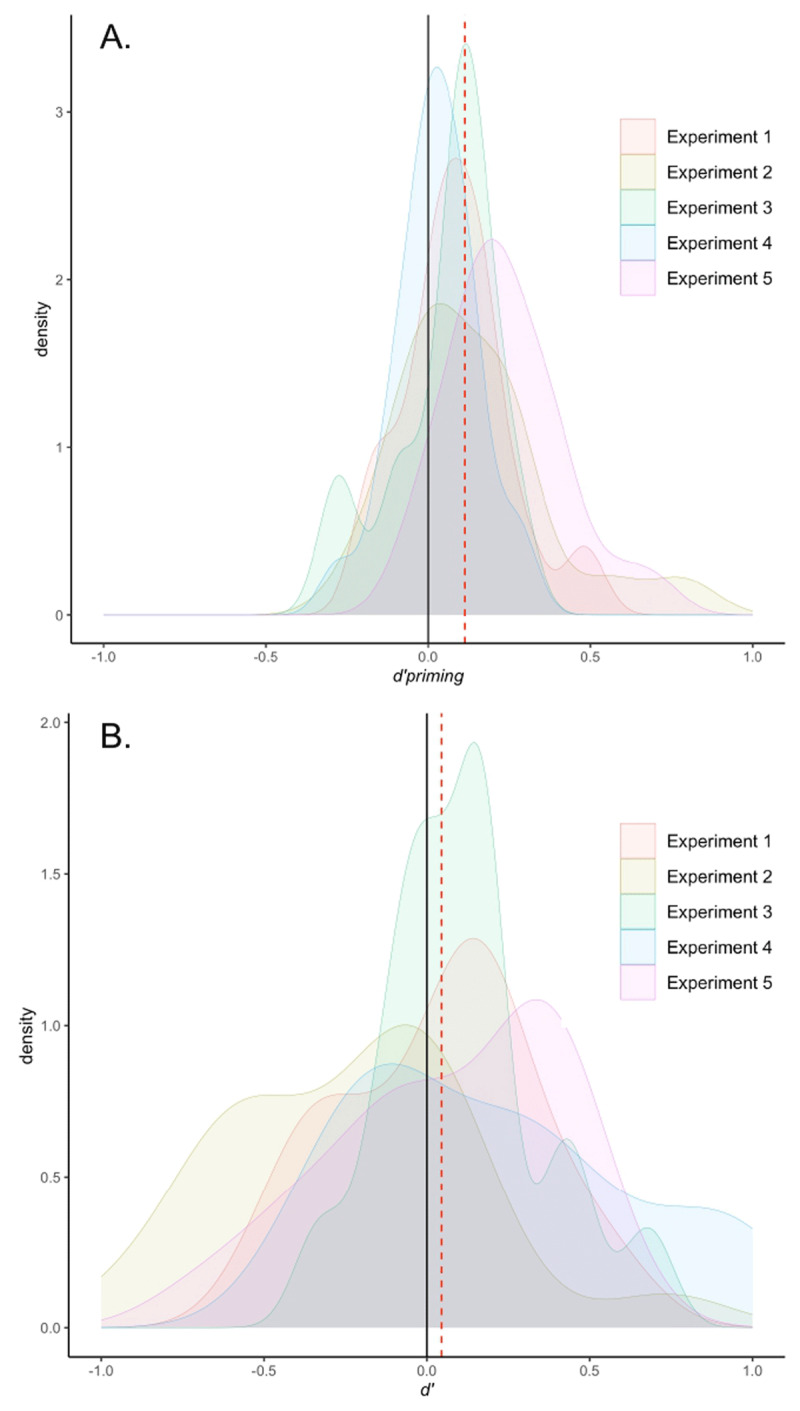
Sensitivity across tasks in Berkovitch and Dehaene’s (2019) dataset. The upper panel (A.) corresponds to the masked priming task. *d*’ _primi__ng_ is plotted across the five experiments. The dotted red line represents the mean *d*’ _priming_ = 0.113. The lower panel (B.) refers to the visibility task for which *d*’ is plotted. The dotted red line represents the mean *d*’ = 0.045.

By recalculating the priming effects as *d*’_priming_ we were able to compare the sensitivity of the masked priming task with the sensitivity of the visibility task (*d*’). In Experiment 1, the comparison between *d*’_priming_ = 0.087 and *d*’ = 0.031 was not significant (*t*_15_ = 0.559, *p* = 0.584). In Experiment 2, the difference between *d*’_priming_ = 0.135 and *d*’ = –0.229 was significant (*t*_18_ = 3.367, *p* = 0.003). The comparison in Experiment 3 was not significant for *d*’_priming_ = 0.055 and *d*’ = 0.114 (*t*_15_ = –0.890, *p* = 0.387). In Experiment 4, the difference between *d*’_priming_ = 0.028 and *d*’ = 0.207 was significant (*t*_23_ = –2.053, *p* = 0.051, but favoring the visibility measure), as well as in Experiment 5 when comparing *d*’_priming_ = 0.236 and *d*’ = 0.064 (*t*_23_ = 2.329, *p* = 0.029). Finally, a statistical contrast combining the five experiments was performed. The mean *d*’_priming_ was 0.113 and the mean *d*’ was 0.045. The comparison did not yield a significant effect (*t*_98_ = 1.554, *p* = 0.123).^4^ Figure 7 depicts the distributions of both *d*’_priming_ and *d*’ for each experiment. The curves represent where the sensitivity scores for priming and visibility are primarily concentrated. In addition to visually confirming that both *d*’ and *d’_priming_* are similarly centered slightly above zero, the figure also reveals a wider distribution of positive and negative values across the entire range for *d*’.

Although these results indicate that the difference between priming and visibility sensitivities is significant in three of the experiments, only Experiments 2 and 5 show evidence of unconscious processing according to this method. While sensitivities in Experiment 4 differed across the two tasks, they did so in the opposite direction. Specifically, participants were more sensitive to the masked primes in the visibility task compared to the priming task. Overall, the results suggest that there is at most only weak evidence that more information from the masked prime is transmitted through behavioral responses (speed of responding to the target stimulus) than through a forced-choice awareness response. As such the results are in alignment with the findings of Meyen et al. (2022). In their reassessment of 15 classic masked priming studies, a similar lack of evidence for greater sensitivity in priming than awareness was observed.

## Discussion

Within the psycholinguistics literature, masked priming has been utilized to draw conclusions regarding the automaticity of syntactic processing (e.g., Jiménez-Ortega, 2014, 2021; Pyatigorskaya, Maran, & Zaccarella, 2023). This line of inquiry follows the logic that if syntax does not require conscious awareness to establish a syntactic relationship between pairs of words, it provides evidence of the automaticity of this higher-order linguistic process. However, this conclusion may be flawed due to methodological limitations. Specifically, this reasoning assumes that masked primes are unconsciously processed, but low statistical power and the low reliability of visibility measures may also contribute to the results (Shanks, Malejka, & Vadillo, 2021). We have addressed these potential issues, demonstrating that consciousness measures in this field suffer from the same problems previously reported in relation to non-linguistic processes. Our results are consistent with there being minimal contribution of unconscious processes to masked syntactic priming, at least in the available set of published studies on this phenomenon. We will now discuss the outcomes according to each of the strategies pursued.

### Chance-Level Visibility

In most of the experiments reviewed in the present article, it was concluded that syntactic processing is automatic or subliminal because masked primes can facilitate or hinder the categorization of targets under conditions where participants seem unable to consciously perceive the prime. In particular, participants’ ability to discriminate the primes’ linguistic category above chance in the visibility test at the end of the experiment is weak (Ansorge et al., 2013; Lijima & Sakai, 2014; Berkovitch & Dehaene 2019). Contrary to this conclusion, a high-powered meta-analysis of participants’ performance in the visibility test revealed above-chance discrimination. Pyatigorskaya, Maran, and Zaccarella (2023) had already reported significant effects in their Pilot 2 and Pilot 3 visibility tests. Based on these results, it is possible to conclude that the other experiments included in the meta-analysis failed to find a significant effect against chance because of a lack of statistical power.^5^ The significant result of the meta-analysis aligns with our prediction and is not surprising since the number of participants tested in these studies is limited, ranging from 6 to 39, as well as the number of trials, which are typically fewer in the visibility task compared to the priming task. Our analyses show that under these conditions, the power to detect a visibility effect like the one observed at the meta-analytic level ranges from 13.1% to 70% for individual studies. To reach a power of 80%, experiments would need to include at least 49 participants, which is far greater than the typical sample size of these studies. It is worth noting that syntactic priming is not unusual in this respect. Experimental research across the whole of psychology has typically suffered from problems related to power (Bakker et al., 2016; Button et al., 2013).

One could argue that although visibility is above chance, it is too low to be considered important (*d*’ = 0.11). Two points need to be stressed regarding this argument. Firstly, the sensitivity in the awareness task is, of course, not expected to be high, since the masking procedure deliberately and significantly reduces the visibility of masked words. Secondly, and more importantly, to evaluate the magnitude of the observed *d*’ it is necessary to compare it with the magnitude of the priming effect, but none of these studies performed this comparison. However, we transformed the priming reaction times of the five experiments performed by Berkovitch and Dehaene (2019) into a scale similar to visibility *d*’, following the sensitivity comparison procedure (Meyen at al., 2022). When considering the results of the individual experiments we found mixed evidence. While two of them revealed more sensitivity for masked priming compared to visibility, one experiment showed the opposite result, and in the other three the comparison was not significant. After collapsing data from all five experiments, we found that visibility scores (*d*’ = 0.045) were not significantly lower than sensitivity scores computed from reaction times in the priming task (*d’_priming_* = 0.113). Therefore, visibility scores are just as small as we should expect them to be, given the size of the priming effect.

The Bayesian framework provides another means to test whether participants were indeed unable to perceive the primes. On the one hand, a Bayesian analysis conducted separately for each experiment yielded mixed evidence, with some favoring and some opposing the hypothesis of conscious awareness. Specifically, the individual BFs for nine experiments support conscious processing of the masked words, while seven show evidence for unconscious processing. However, the evidence in favor of the conscious hypothesis received strong support from four experiments, while the remainder revealed at best anecdotal or moderate support. This scenario highlights why interpreting null results in NHST as indicative of the null hypothesis is unjustified (Dienes, 2014; Shanks, Malejka, & Vadillo, 2021; Vadillo, Konstantinidis, & Shanks, 2016) and can lead to erroneous conclusions, as evidenced in this context regarding the automaticity of syntactic linguistic processing.

The experiments showing strong evidence for conscious processing are Pilot 3 and the three main experiments by Pyatigorskaya, Maran and Zaccarella (2023). Interestingly, these last three have the largest samples and the highest statistical power. This supports our thesis that strictly methodological reasons are driving the null outcomes of visibility tests reported in the reviewed studies. Furthermore, even if the disparity of results found in the Bayesian analyses across experiments reflects the possibility of doubt, the result of the Bayesian meta-analysis, which considers all sixteen experiments together to compute a unique BF, shows extreme evidence for the visibility of the masked words. It is not surprising that this overall result differs from the BF of some of the individual experiments due to the weighting process, favoring those with larger sample sizes and lower variance. Therefore, consistent with the results of the frequentist meta-analysis, the Bayesian meta-analysis suggests that participants could see the masked words despite the fact that they were expected to be subliminal.

### Non-Significant Correlation Between Visibility and Priming

A second argument to claim that syntactic priming is unconscious is that the size of syntactic priming does not correlate with prime visibility; that is, participants who seem more capable of perceiving the primes are not the ones that show the strongest priming effects. But, as claimed elsewhere (Vadillo et al., 2022), a lack of correlation between dependent measures might be attributable to correlation attenuation due to their limited reliability.

Our reanalysis of the data gathered by Berkovitch and Dehaene (2019) revealed that the split-half reliability of masked priming was remarkably low across the five experiments. Even after correcting negative estimates, the average r*_xx_ remained lower than 0.1 in the first four experiments, increasing to 0.5 in Experiment 5. In the case of the visibility task, reliability scores were also low, ranging from 0.034 in Experiment 3 to 0.484 in Experiment 4, where the visibility task reached the highest r*_xx_.

Contrary to our expectations, before the correction, mean *r_xx_* was negative in many of the experiments and within both tasks, although it was particularly characteristic of the masked priming task. While reliability is generally anticipated to fall within the range of 0.0 to 1.0, this isn’t always the case in practice. That is to say, while these results are unexpected, they are not unprecedented. For instance, Pronk et al. (2021) analyzed four independent datasets from different experimental tasks and reported a negative split-half reliability in one of them. Similarly, Yaron et al. (2024) recently calculated the reliability of awareness measures from 18 datasets, and 7 of them showed negative reliability scores. In their seminal paper ‘A note on negative reliabilities’, Cronbach and Hartman (1954) posit that one potential explanation for negative reliabilities is the imperfect balance of split halves derived from the test. In our study, we addressed this limitation by stratifying the data when dividing it into halves during each permutation. Such a stratification procedure, recommended in reliability calculations (Pronk et al., 2021), ensured that each half contained an equal number of elements from every block, as well as from each prime and target type, and trial congruency; thus mitigating any potential effects on performance. Negative reliabilities can also arise if the data do not comply with the assumption of equal covariances within each half (Cronbach & Hartman, 1954). Another potential explanation for negative reliabilities is specific to difference scores, which is particularly relevant in this case since the priming effect is essentially a difference score of reaction times in the categorization task between incongruent and congruent trials. This phenomenon occurs when the correlation between both elements of the difference score is high, resulting in low variance and, consequently, reliability close to zero (Parsons, Kruijt, & Fox, 2019). However, the lack of precision in the estimation can make the correlation appear negative. This explanation is consistent with the reduced number of observations included on each half for calculation of the reliability, with an average of 29 trials per participant in the case of the *d*’ measure.

Regardless of the specific reasons for our negative reliability estimates, it seems safe to conclude that the reliabilities of masked priming in four of the five experiments can be interpreted as being essentially zero, and three of the visibility tasks also show reliabilities around 0.1 or zero, with the other two being positive but low. The low number of trials might explain the low reliability, particularly in the visibility task (40–64 trials across experiments). Recent simulation studies suggest that at least 200 trials are necessary to obtain reliable measures, although this depends on variability in awareness across participants (Yaron et al., 2024). Given the observed measurement error present in these tasks, it would have been surprising to find a significant correlation between the priming effect and the visibility score. According to the results from the psychometric meta-analysis, which accounts for this measurement error in the calculation of the correlations, the confidence interval for the estimated correlation spans the entire range of possible values from –1 to 1. This means we cannot draw any conclusions about this correlation or what it implies about the relationship between priming and awareness at the latent level.

### Recommendations for Future Research

The ultimate goal of this work is not to criticize the methodological problems of a specific study per se, but to highlight and discuss the methodological pitfalls that may be repeatedly present in masked syntactic priming research. We believe in the common interest of psycholinguistics in improving its methods to generate knowledge that reflects reality as accurately as possible. Therefore, we use the previously discussed limitations to propose some suggestions that can be applied in future research, in line with those made by Shanks, Malejka, and Vadillo (2021).

Given that some of the inferences drawn in this literature hinge critically on statistical power, we advocate both for the utilization of larger sample sizes (Button et al., 2013) and the inclusion of more trials per condition (Baker et al., 2021; Biafora & Schmidt, 2019). According to the studies included in the frequentist meta-analysis, we estimated that 49 participants would be necessary to reach statistical power of .80. However, power analyses need to be conducted a priori case-by-case. Recognizing the challenges researchers encounter in participant recruitment, conducting a single experiment with a substantial sample size may yield greater benefits compared to allocating resources across numerous experiments with smaller samples.

Additionally, whenever inferences are based on correlational evidence, it is imperative to calculate and report the reliability of both the priming and visibility tasks. The number of observations seems a critical feature to consider. In the example dataset used here, Berkovitch and Dehaene (2019) included a variable number of masked trials, either 240 or 480 (Exp. 4 and 5) in the priming task and ranging from 40 to 64 in the visibility task, which we demonstrated are insufficient for acceptable reliability rates. Simulation studies in other paradigms show that with a sufficiently large number of experimental trials, reliability could progressively approach 1.0 (Vadillo et al., 2022; Yaron et al., 2024). However, it is essential to note that this may not always be the case, as other studies using this method did not achieve the desired result (Enkavi et al., 2019).

Finally, if the aim is to investigate a null effect, it is advisable to leverage the Bayesian framework to seek evidence for the null hypothesis (Dienes, 2014; Shanks, Malejka, & Vadillo, 2021). Moreover, the use of structural equation models has been proposed to study latent unconscious processing, along with the application of linear mixed models, which can be more robust in addressing issues related to the limited number of trials (Hedge, Powell, & Sumner, 2018).

In a recent article, Michel (2023) drew a comparison between research in consciousness and the Sisyphus metaphor, suggesting that just when it seems the field has made progress, new criticisms emerge, reversing the advances. Considering that the utilization of masked priming to investigate syntactic processing using objective consciousness measures has primarily flourished within the last decade, we still maintain a positive outlook and believe it is not too late to refine our research practices and construct a robust knowledge foundation. This may be especially crucial in the realm of syntax compared to other forms of masked linguistic priming due to the far-reaching implications of its findings, as syntax has been suggested as the core of human language (Fitch, 2014; Hauser, Chomsky & Fitch, 2002; Zaccarella & Friederici, 2017).

Our results challenge previous assumptions derived from masked priming effects supporting the automatic nature of syntactic processing. This criticism does not necessarily extend to other sources of evidence for unconscious syntactic processing. Hence, experimental paradigms such as rapid serial visual presentation or continuous flash suppression deserve a critical analysis of their own. Although engaging in a debate on the computational architecture of language is not the primary focus of the present paper, we highlight the importance of entering this intellectual discourse equipped with the appropriate tools. Otherwise, conclusions drawn from weak methodological procedures may not even require additional empirical evidence to be dismissed, they would simply be meaningless.

## Data Accessibility Statement

The analysis scripts are available online through the Open Science Framework (https://osf.io/85rjd/) to facilitate the application of these procedures in future research within the field. Data for the meta-analysis can be found in Table 2. The remaining data belong to Berkovitch and Dehaene (2019) and therefore have not been made available.

## Footnotes

Based on the recommendation of an anonymous reviewer, we also repeated the search using different terms with the following string: ‘(syntax or syntactic or morphosyntactic) and (nonconscious or subconscious)’. This returned only one study that was not retrieved in the original search, but it was ultimately found to be ineligible.

At the time of establishing the inclusion criteria, we did not account for the possibility that awareness of the primes could be measured during the main priming task, as pointed out by an anonymous reviewer. However, this did not affect the selection process, as no studies were excluded based on this factor.

The values provided correspond to our reanalysis, not to the original values reported by Berkovitch and Dehaene (2019), which are summarized in Table 1.

An anonymous reviewer raised the possibility that computing the sensitivity parameter *d_priming_* would reduce the sensitivity of the priming task by dichotomizing the originally continuous priming scores in milliseconds. In the end, this could bias the comparison with the sensitivity in the visibility task (*d*’). To test this possibility, we collapsed the five experiments, compared both priming and *d_priming_* scores against zero and calculated the standardized effect size (Cohen’s *d_z_*) in each case. If sensitivity had been reduced, the effect size would be lower in the *d_priming_* contrast than the priming one. However, our results revealed a very similar Cohen’s *d_z_* in both cases, equal to 0.63 for the priming contrast and 0.59 for the dichotomized *d’_priming_* contrast. Therefore, there is no evidence that the Meyen method (Meyen et al., 2022), which allows us to compare the priming task and the visibility task, is biased for this reason, at least in the present data.

It should be noted that Pyatigorskaya, Maran, and Zaccarella (2023) did not test against zero visibility in their three main experiments, which had the highest statistical power, ranging between 60% and 70%. Their motivation was to replicate the masked priming effects observed by Berkovitch and Dehaene (2019) in their Experiment 3. To ensure comparability across studies, the authors contrasted their participants’ *d*’ scores against 0.12, the non-significant *d*’ score reported by Berkovitch and Dehaene in that specific experiment. Additionally, they also relied on the null correlation strategy to argue that prime visibility did not predict performance in the masked priming task.
